# Hepatoid adenocarcinoma of the stomach: CT findings

**DOI:** 10.3389/fonc.2023.1036763

**Published:** 2023-02-03

**Authors:** Qian Yang, Yulin Liu, Shuixia Zhang

**Affiliations:** Department of Radiology, Hubei Cancer Hospital, Tongji Medical College, Huazhong University of Science and Technology, Wuhan, China

**Keywords:** PVTT: portal vein tumor thrombus, hepatoid adenocarcinoma of the stomach, computed tomography scan, metastasis, AFP

## Abstract

**Objective:**

To analyze the CT findings of hepatoid adenocarcinoma of the stomach (HAS) and improve the diagnosis accuracy of this condition.

**Methods:**

The CT images of 22 pathologically confirmed HAS patients were analyzed retrospectively. We investigated the location of lesions, morphology, enhancement features, area of invasion into surrounding organs, lymph node metastasis, and venous tumor thrombus.

**Results:**

Among the 22 patients (17 men and 5 women, the mean age was 61.41 ± 9.83 years ranging from 36 to 80 years) with HAS; the morphology of tumors included mass (n = 5), focal ulcer (n = 7), and infiltrating ulcer (n = 10). Extraserous fat was invaded in 12 cases. Enhancement scans showed continuous enhancement in all cases. The CT values of unenhanced scan, the arterial phase, and the portal venous phase are 30.36 ± 6.46, 60.91 ± 17.80, and 75.64 ± 22.09 (Hounsfield Unit, HU), respectively. In six cases, the tumor infiltrated the surrounding organs: liver (n = 1), pancreas (n = 2), and both liver and pancreas (n = 3). In 16 out of 22 patients (72.3%), suspicious lymph node metastasis at CT imaging has then been confirmed by pathological specimens. Intrahepatic metastasis was found in 14 cases. Seven patients had venous tumor thrombus: three patients developed tumor thrombus in the main trunk and intrahepatic branches of the portal vein and two patients in the portal vein, splenic vein, and superior mesenteric vein simultaneously.

**Conclusion:**

The CT scans of HAS often show a thickened gastric wall and infiltrating ulceration. Infiltration into extraserosal fat is often seen. Enhancement scans show a continuous and progressive enhancement of lesions. Lymph node metastasis, intrahepatic metastasis, and portal vein tumor thrombus are common in HAS patients.

## Introduction

Hepatoid adenocarcinoma of the stomach (HAS) is a special category of gastric cancer that presents the differentiation characteristics of both adenocarcinoma and hepatocellular carcinoma. It is a rare type of gastric cancer, accounting for 0.38%–1.6% of all gastric cancers (1–3). HAS is most often diagnosed through pathomorphology, and most patients have elevated serum alpha-fetoprotein (AFP) levels. The prognosis of HAS is worse than common gastric cancers (1–3). Hepatoid adenocarcinoma is most often seen in the stomach (63%) and ovaries (10%). It is also reported in other organs such as lungs (5%), gall bladder (4%), pancreas (4%), uterus (4%), bladder (4%), esophagus (1%), and colon (1%) (4–8). “Hepatoid adenocarcinoma of the stomach” was first reported and named by Ishikura et al. in 1986. The frequent misdiagnosis of HAS is due to limited knowledge about its clinical and pathological features. Little is reported on the imaging features of HAS (9–11). This paper retrospectively analyzes the CT findings of 22 patients with an aim to improve the diagnosis of HAS.

## Materials and methods

We retrieved 25 cases with HAS from pathology and radiology records and clinical records between January 2014 and December 2020. Among these patients, 22 were pathologically confirmed and had enhancement CT images of the upper abdomen, and 3 had no abdominal enhancement CT images. All patients were followed up after treatment at our center. The diagnosis of HAS was based on pathomorphological evaluation. The gastric cancer patients found with hepatoid differentiation were diagnosed as HAS. This study was approved by the ethical committee, and informed consent was obtained.

### CT scans

All patients were on preoperative fasting 8 h prior to examination and were given 500–800 ml of water within 30 min at the time of examination. Patients were scanned from the right diaphragmatic dome to the iliac crest with multislice spiral CT (GELightSpeed VCTor SOMATOM Definition). For CT values, the tube voltage is 120 kV. The tube current is 220–280 mA. The matrix is 512 × 512 continuous scanning without space. Slice thickness was 5 mm, while reconstruction thickness was 3.0 mm. Iopamidol (370 mg I/ml) was injected through a high-pressure syringe into peripheral veins as a contrast agent at 1.5 ml/kg dose and 2.5–3.5 ml/s flow rate. After injection, all patients were examined for 30–40 and 60–80 s with arterial phase and parenchymal phase scanning while holding breath. All images were transmitted to the working station through the picture archiving and communication system for further analysis and measurements.

### Image analysis

All patients’ data were reconstructed on sagittal and coronal planes and analyzed by two experienced radiologists for (1) tumor morphology (according to the Bormann standard:nodule or mass, focal ulcer, infiltrating ulcer, and diffuse thickening). (2) Tumor density: the CT attenuation values of gastric lesions and/or hepatic tumor lesions (the largest intrahepatic lesion) on unenhanced CT scans, the enhanced arterial phase, and the venous phase were measured three times and averaged, avoiding tumor necrosis areas (9). (3) Infiltrating depth into the gastric wall and whether adjacent organs (pancreas/liver) were invaded. (4) Lymph node metastasis: We marked the location and number of lymph nodes and evaluated them based on short axis measurement. Lymph node metastasis was defined as a short axis diameter ≥ 8 mm with inhomogeneous enhancement or >3 small lymph nodes within a single group (10). (5) The imaging features of other abdominal organs, for instance, hepatic metastasis, or the existence of tumor thrombus in the portal vein and perigastric veins (11).

### Statistical methods

SPSS 21.0 software was used for statistical analysis. Measurement data that conform to normal distribution were indicated as mean ± SD, and intergroup comparison was carried out through an independent two-sample t-test. The CT values of subgroups were compared using a t-test. For non-parametric distributions, the chi-square test was used for intergroup comparison, and *P* < 0.05 was defined as a statistically significant difference.

## Results

Of the 22 patients in this study, including 17 men and 5 women, the mean age was 61.41 ± 9.83 years ranging from 36 to 80 years. There were 19 patients who had abdominal discomfort, 2 had melena and hematemesis, and 1 had no obvious clinical symptoms. A total of 14 patients had elevated serum AFP (>5.5 IU/ml), 15 had elevated carcinoma embryonic antigen (CEA), and 9 had elevated carbohydrate antigen 199. No other cause for AFP elevation was found such as hepatitis or cirrhosis. All clinical data are shown in **Table 1**.

**Table 1:** Clinical data and image findings of 22 patients.

No./Gender/Age	Serum AFP(IU/ml)	CA199(μ/ml)	CEA(μg/L)	Location	Morphology	Image findings of gastric lesions	Maximum short diameter of metastatic LN(cm)	Distant metastases	Adjacent organ invasion	Tumor thrombosis
1/F/49	777.30	Normal	Elevated	Stomach body	Infiltrating ulcer	Thickened gastric wall with ulcer, serosal infiltration, and inhomogeneous enhancement	2.9	Multiple lymph node metastasis at the hepatic hilum and retroperitoneal area	Pancreas and liver	Main trunk and intrahepatic branches of portal vein, splenic, vein and SMV
2/F/67	362.48	Normal	Elevated	Gastric antrum	Mass	Thickened gastric wall without serosal infiltration. Inhomogeneous enhancement	1.5	Liver	Liver and Pancreas	None
3/F/51	113.30	Elevated	Elevated	Gastric antrum	Focal ulcer	Partially thickened gastric wall. No serosal infiltration. Homogeneous enhancement	0.8	Liver	None	None
4/M/66	159,855.9	Elevated	Normal	Stomach body	Infiltrating ulcer	Thickened gastric wall with ulceration. Serosal infiltration. Inhomogeneous enhancement	1.8	Liver	None	Emboli inside left branch of portal vein and its branches and the right anterior branch of the portal vein
5/M/64	2.29	Elevated	Elevated	Stomach body	Mass	Lumpy thickening of gastric wall. Serosal infiltration. Inhomogeneous enhancement	1.0	None	Crus of diaphragm, adrenal gland, and pancreas	None
6/M/47	439	Normal	Normal	Gastric antrum	Focal ulcer	Partially thickened gastric wall. No serosal infiltration. Inhomogeneous enhancement	0.8	Liver	None	Emboli inside right gastroepiploic vein
7/F/61	1.85	Elevated	Elevated	Cardia	Infiltrating ulcer	Thickened gastric wall with ulceration. Serosal infiltration. Inhomogeneous enhancement	1.6	None	None	None
8/M/63	2.34	Normal	Elevated	Gastric antrum	Infiltrating ulcer	Thickened gastric wall with ulceration. Serosal infiltration., Inhomogeneous enhancement	——	Liver	None	None
9/M/54	1,033.25	Normal	Elevated	Stomach body	Infiltrating ulcer	Thickened gastric wall with ulceration. Serosal infiltration., Inhomogeneous enhancement	2.0	Liver	None	None
10/M/72	19,460	Normal	Normal	Gastric antrum	Infiltrating ulcer	Thickened gastric wall with ulceration. Serosal infiltration. Inhomogeneous enhancement	1.8	Liver	None	None
11/M/55	746.7	Normal	Elevated	Gastric antrum	Mass	Thickened gastric wall. No serosal infiltration. Inhomogeneous enhancement	1.2	Liver	Liver	None
12/M/66	8.46	Normal	Elevated	Stomach body	Infiltrating ulcer	Thickened gastric wall with ulceration. Serosal infiltration. Inhomogeneous enhancement	——	None	None	None
13/M/36	56.7	Normal	Normal	Stomach body	Focal ulcer	Partially thickened gastric wall. No serosal infiltration. Inhomogeneous enhancement	——	None	None	None
14/M/59	11.21	Normal	Elevated	Stomach body	Focal ulcer	Partially thickened gastric wall. No serosal infiltration. Homogeneous enhancement	——	None	None	None
15/M/64	2.06	Elevated	Elevated	Stomach body	Infiltrating ulcer	Thickened gastric wall with ulceration. Serosal infiltration. Inhomogeneous enhancement	——	Liver	None	Left branch of the portal vein
16/F/72	2.92	Normal	Normal	Stomach body	Focal ulcer	Partially thickened gastric wall. No serosal infiltration. Homogeneous enhancement	0.9	Liver	None	None
17/M/80	375.4	Elevated	Normal	Gastric antrum	Mass	Thickened gastric wall. No serosal infiltration. Inhomogeneous enhancement	0.9	None	Descending duodenum and head of pancreas	Superior mesenteric vein (SMV)
18/M/67	4.60	Elevated	Elevated	Gastric antrum	Focal ulcer	Partially thickened gastric wall. No serosal infiltration. Homogenous enhancement	1.2	Liver	None	None
19/M/72	489.40	Normal	Normal	Cardia	Mass	Thickened gastric wall. Serosal infiltration. Inhomogeneous enhancement	1.8	Liver	Liver, Pancreas	Portal vein, splenic vein, and SMV
20/M/60	3.27	Elevated	Elevated	Stomach body	Focal ulcer	Partially thickened gastric wall. No serosal infiltration. Homogeneous enhancement	1.0	Liver	None	None
21/M/64	118.0	Elevated	Elevated	Cardia	Infiltrating ulcer	Thickened gastric wall with ulceration. Serosal infiltration. Inhomogeneous enhancement	2.3	Liver	None	Portal vein
22/M/62	1.6	Normal	Elevated	Gastric fundus	Infiltrating ulcer	Thickened gastric wall with ulceration. Serosal infiltration. Inhomogeneous enhancement	——	Liver	None	None

All patients had gastroscope and tissue biopsy. There were 16 patients who had gastrectomy; 2 had exploratory laparotomy with enterolysis, gastrojejunal bypass, and enteroanastomosis; 1 had hepatic tumor excision without gastric surgery; and the remaining 3 patients were not treated with surgery. All 22 patients received pre- or postoperative chemotherapy.

### Histopathological results

The mean length of tumor was 9.8 cm (4.5–15.0 cm). Pathological staging of the 16 patients with gastrectomy were as follows: 1 case at the T1 stage with a tumor in the mucosal layer; 9 cases at the T3 stage with tumorpenetrating subserosal connective tissue but not invading visceral peritoneum or adjacent structures; and 6 cases at the T4 stage with tumor-infiltrating serosa (visceral peritoneum) or adjacent structures. There were 12 patients who had immunohistochemical AFP staining; 11 were positive, and 1 was negative (**Table 1**).

### CT findings

There were 9 cases out of the 22 that had lesions in the gastric antrum, 10 in the gastric body, and 3 at cardia, and gastric wall thickening was seen in all cases. There were 12 patients who had serosal infiltration, and morphological findings include mass (n = 5), focal ulcer (n = 7), and infiltrating ulcer (n = 10). The mean CT attenuation values of unenhanced scans, the arterial phase, and the portal venous phase were 30.36 ± 6.46, 60.91 ± 17.80, and 75.64 ± 22.09 HU, respectively. The CT value of the venous phase was 14.73 ± 8.98 HU (*t* = 7.70, *P* = 0.00) higher than that of the arterial phase, and continuous enhancement was seen in all cases. There were 5 out of 22 cases that showed homogeneous enhancement, and 17 showed inhomogeneous enhancement. No significant difference was noted on gastric lesion enhancement CT values between the group with elevated serum AFP and the group with normal serum AFP and between the groups with or without gastric serosal infiltration. The CT difference (namely, ΔCT) between enhanced CT and plain CT had no significant difference (**Table 2**). For adjacent organ invasion, four had infiltration in the liver and five in the pancreas (among which three had invasion in the liver and pancreas simultaneously). A total of 16 patients were found with lymph node metastasis on CT (a short diameter of the lymph node ≥ 8 mm), The accuracy of the CT diagnosis of LN metastasis was 72.7% (16/22). All had perigastric lymph nodes; one had retroperitoneal lymph nodes. The short diameter of the largest lymph node was 1.11 ± 0.80 cm, and all of them were confirmed with lymph node metastasis postoperatively. Two patients were not found with lymphadenectasis on CT but were found with lymph node metastasis in pathological results.

**Table 2:** CT values of a gastric tumor.

CT value (HU)	Gastric Tumor				Gastric Tumor				Gastric Tumor			
	Liver Metastasis(n = 14)	No Liver Metastasis(n = 8)	t	P	Elevated AFP(n = 14)	Normal AFP(n = 8)	t	P	Serosal Infiltration(n = 12)	No Serosal Infiltration(n = 10)	t	P
Plain CT	30.27 ± 6.63	30.00 ± 6.93	0.195	0.847	29.29 ± 6.83	32.25 ± 5.62	1.038	0.312	29.50 ± 6.01	31.40 ± 7.14	0.679	0.505
Arterial phase	61.64 ± 19.46	59.63 ± 15.62	0.250	0.805	57.29 ± 14.11	67.25 ± 22.55	1.282	0.214	58.17 ± 11.46	64.20 ± 23.59	0.784	0.442
Venous phase	75.36 ± 25.58	76.13 ± 15.77	0.077	0.940	70.93 ± 16.73	83.88 ± 28.68	1.348	0.193	69.25 ± 13.10	83.30 ± 28.45	1.532	0.141
ΔCT(A-P)^*^	29.79 ± 17.24	27.88 ± 8.61	0.291	0.774	25.71 ± 9.47	35.00 ± 19.99	1.488	0.152	26.17 ± 10.73	32.60 ± 17.98	1.040	0.311
ΔCT(V-P)^*^	43.36 ± 23.51	45.5 ± 10.74	0.242	0.811	39.86 ± 14.60	51.63 ± 25.48	1.388	0.180	38.08 ± 12.93	51.40 ± 24.07	1.656	0.113

“ * “ stands for the CT difference (namely, ΔCT) between enhanced CT and plain CT.

**Figure 1:**
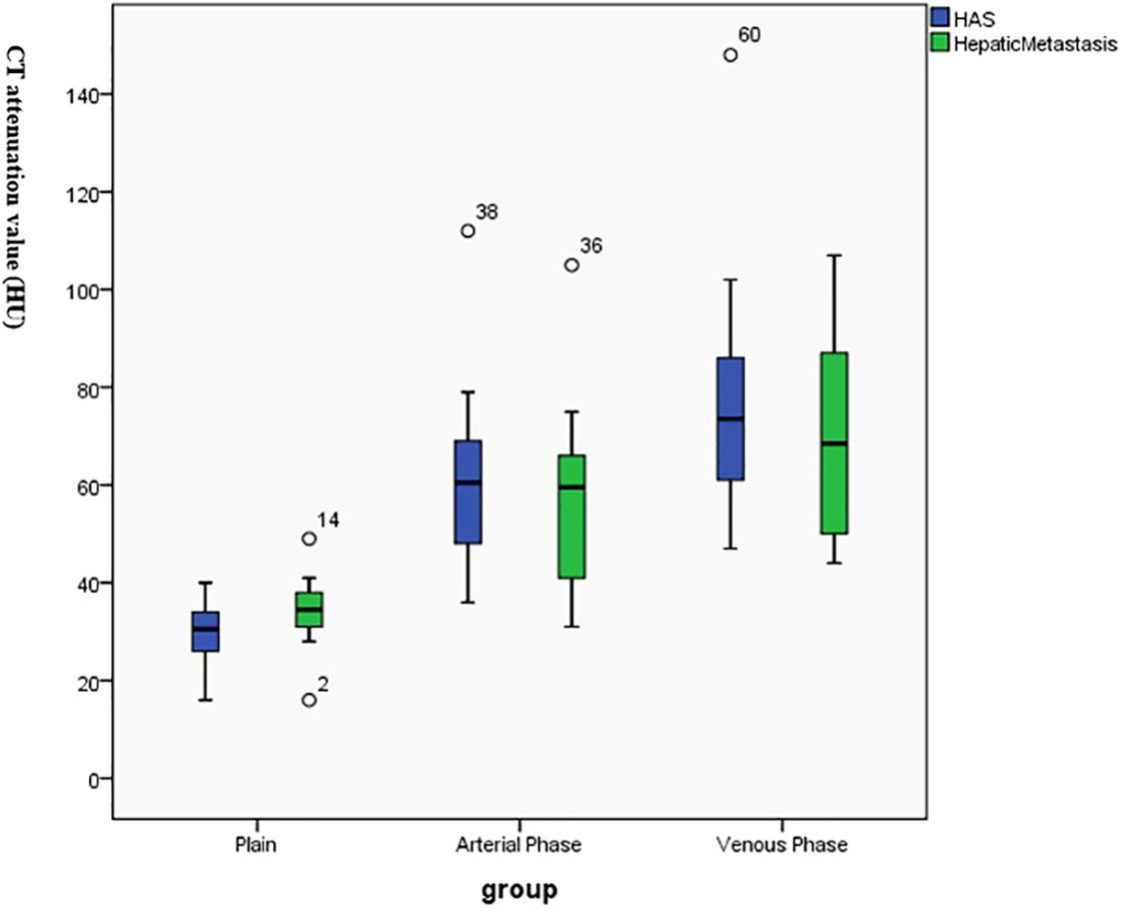
Continuous enhancement of hepatoid adenocarcinoma of the stomach (HAS) and hepatic metastatic lesions on enhancement CT.

**Figure 2:**
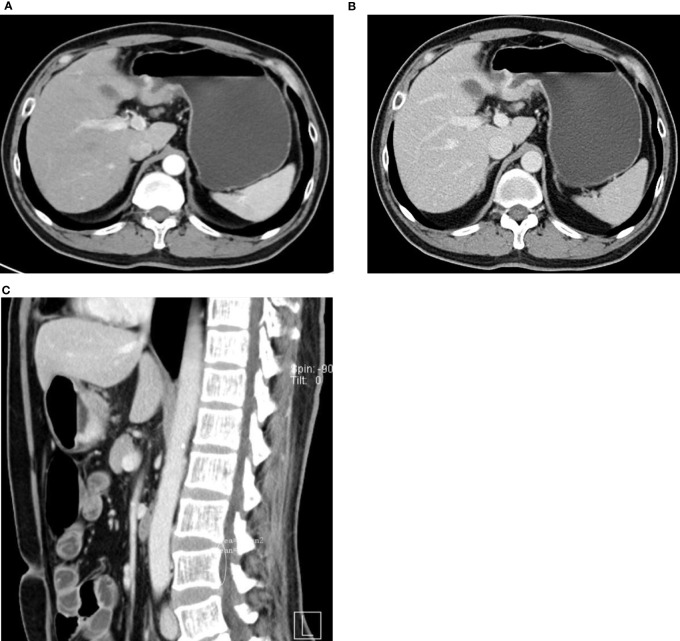
47-year-old man with HAS. **(A–C)** Thickened wall at the gastric antrum. Nodular/irregular protrusion with an obscure surrounding fat layer. Evident continuous enhancement of lesion on enhancement CT.

**Figure 3:**
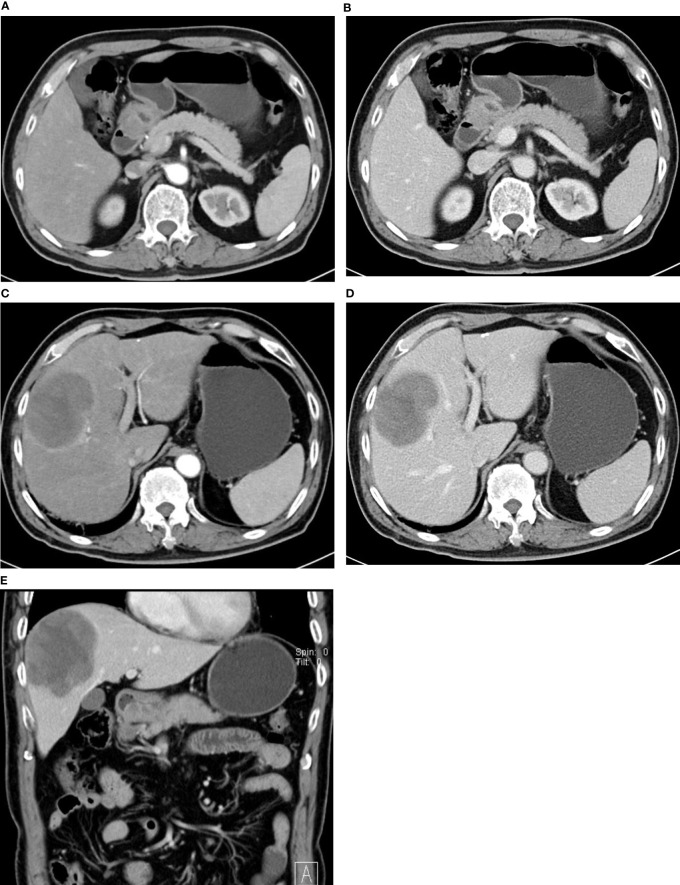
72-year-old man with HAS. Thickened wall at the gastric antrum with infiltrating ulceration. Continuous mild-to-moderate enhancement in the arterial phase **(A)** and venous phase **(B)**. **(C, D)** shows continuous a mild-to-moderate enhancement of mass in the right hepatic lobe in the arterial phase and portal venous phase. Sagittal view **(E)** shows ulceration at the gastric antrum and metastatic lesions in the right lobe.

There were 14 patients who were found with hepatic metastatic tumors; 6 of them had multiple intrahepatic metastasis manifested as slightly lower-density lesions with unclear margins and inhomogeneous density on imaging. Metastatic lesions were consistent with primary lesions (**Table 3**) as continuous progressive enhancement (**Figures 1**–**3**).

**Table 3:** CT value comparison between hepatic metastasis lesions and primary gastric lesions in the hepatic metastasis group.

	Plain CT(HU)	Arterial Phase(HU)	Venous Phase(HU)	ΔCT(A-P)(HU)	ΔCT(V-P)(HU)
Gastric lesion	30.57 ± 6.43	61.64 ± 19.46	75.36 ± 25.58	29.79 ± 17.24	43.36 ± 23.51
Hepatic lesion	34.07 ± 7.50	57.36 ± 20.09	69.86 ± 20.37	23.5 ± 15.11	35.79 ± 17.13
***t***	1.326	0.573	0.629	1.026	0.974
***P***	0.196	0.571	0.535	0.314	0.339

**Figure 4:**
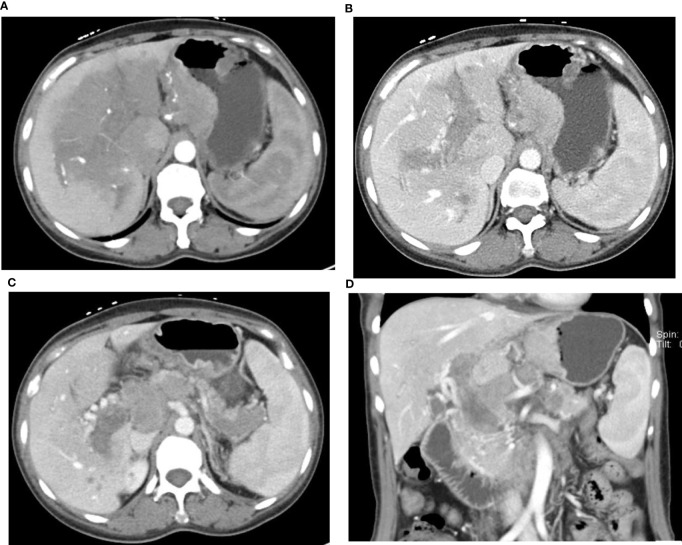
49-year-old woman with HAS and portal vein tumor thrombus. AFP: 777.30 IU/ml. The mass at the lesser gastric curvature shows mild-to-moderate enhancement in the arterial phase **(A)** and venous phase **(B)**. Large area of low density in the hilar parenchyma with arterial phase enhancement lower than normal parenchyma. Tumor thrombus in the portal vein and splenic vein, Collateral circulation formed around the portal vein **(C, D)**, Multiple LN metastasis in the perigastric area, behind the hepatic hilum—the head of pancreas, and the retroperitoneal area **(C, D)**.

Seven patients (31.8%, 7/22) were found with venous tumor thrombus, five of them at the portal vein, one at the SMV, and one at the right gastroepiploic vein. Portal venous–phase CT showed filling defects inside the portal vein or relevant veins (**Figure 4**). In these seven patients, five had hepatic metastasis and six had elevated serum AFP.

## Discussions

HAS is a rare epithelium-derived malignant neoplasm most often seen in the senior male population, with the morphological features of hepatocellular carcinoma and adenocarcinoma. Elevated AFP is often seen in HAS patients. Clinical symptoms include abdominal pain, abdominal distention, and melena, and none of them is specific to HAS. The mechanism of HAS is not yet clear. It may be because the stomach and liver both originate from the endoderm during embryonic development and were both developed from the primitive foregut. During oncogenesis, some primary gastric tumor cells undergo anomaly differentiation and developed hepatocellular morphology. The hepatocellular carcinoma area and adenocarcinoma area migrate into each other and result in hepatoid adenocarcinoma (12). Hepatoid morphology is an independent factor of a poor prognosis in gastric cancer patients (13).

Neoplastic gastric mucosal cells that differentiated into hepatic cells can produce some substances that normal or neoplastic hepatocytes can produce, including albumin and AFP. In this study, 63.6% (14/22) of HAS patients had elevated serum AFP, and 68.2% (15/22) had elevated CEA. Elevated AFP is seen in most HAS patients, but there are also reports of normal AFP levels (2, 14). Therefore, HAS should be diagnosed based on histological features instead of AFP levels. Some gastric adenocarcinoma patients also had elevated AFP, called AFP-producing gastric cancer (AFPGC). AFPGC is divided into three histological subtypes: hepatoid, enteroblast, and yolk-sac-like subtypes. AFPGC cases are not all hepatoid. Thus, AFP levels alone cannot confirm the diagnosis of HAS. Patients should be diagnosed with HAS when hepatoid morphology is detected in pathological results, with or without AFP generation. In the study of Díez Redondo P et al. (3), 85%–95% of HAS patients had positive AFP results in the IHC staining test and 70%–80% of HAS patients had elevated serum AFP. In this study, the positive rate of AFP staining was 92% (11/12), and the rate of elevated serum AFP was 64% (14/22), which was basically consistent with the report. They believed that AFP levels are correlated with the levels of tumor differentiation. Some patients had elevated serum AFP, indicating higher levels of differentiation in the hepatoid tumor area and a worse prognosis.

In this study, we analyzed the CT findings of 22 HAS patients. Similar to common gastric adenocarcinoma, HAS lesions are often seen in the gastric antrum and gastric body; 77.3% of lesions are focal ulcers or infiltrating ulcers. Serosal infiltration is seen in 54.5% of cases, indicating a strong local infiltrating feature, which echoes the study of Díez Redondo P et al. (3). Variances in the enhancement patterns of gastric lesions on imaging may indicate the blood supply and biological features of the tumor mass. In the study of Choi J et al. (10) on common advanced gastric cancer and enhancement patterns in the three phases, they discovered that most tumors with high and medium differentiation had strong enhancement in the arterial phase, peaking in the venous phase, and slowly subsiding in the equilibrium phase, while some adenocarcinomas with low differentiation showed progressive enhancement. In the study of Ren A et al. (9)on HAS, dynamic scan indicated rapid intensification during the arterial phase followed by continuous progressive enhancement. In this study, 22 HAS patients showed continuous progressive enhancement from the arterial phase to the venous phase on enhancement CT. CT values in the portal venous phase were 3–36 HU higher than arterial phase, which is similar with diffuse gastric cancer in Lauren classification. In this study, there was no evidence that the CT enhancement pattern of gastric lesions in HAS is correlated with AFP elevation, extraserosal invasion, and the existence of hepatic metastasis.

The lymph node metastasis rate of common gastric cancer is 12.5% (15), while, in this study on HAS, the lymph node metastasis rate was 81.8% (18/22). The accuracy of the CT diagnosis of LN metastasis was 72.7% (16/22). The short diameter of the largest LN was 2.9 cm. LN ≥ 0.8 mm was seen in 16 cases, and metastasis was found during LN dissection in two cases with a short diameter <8 mm. Compared with common gastric cancer, HAS has a higher incidence of LN metastasis. In HAS, LN metastatic lesions are larger and more susceptible to necrosis, and enhancement levels are higher in metastatic lesions than in primary lesions (9).

HAS has a strong local invasion property and shows a strong tendency of hepatic metastasis and venous invasion (3, 14, 16). In gastric adenocarcinoma, the hepatic metastasis rate at diagnosis is 2.5% (1), while, in this study on HAS, the hepatic metastasis rate at diagnosis was 63.6% (14/22). Six patients had multiple lesions of various sizes in CT scans, and eight patients had single intrahepatic mass. The enhancement pattern of HAS hepatic metastatic lesions is continuous enhancement, distinctive from the “fast-in and fast-out” pattern of liver cancer (**Figure 3**). Some studies indicated that the hepatotropic property may be correlated with the c-Met gene (1). Met protein is the receptor of the hepatocellular growth factor that is more often expressed in advanced metastatic lesions.

Portal vein tumor thrombus (PVTT) is rare in GI cancers, with an incidence of 1.2% and 0.6% in gastric cancer and colorectal cancer, respectively (17). However, among all HAS patients in this study, this incidence was 31.8% (7/22). Araki et al. (18) reported the correlation between gastric cancer with PVTT and elevated AFP. Terracciano et al. (1) reported evident vascular invasion in pathological results in all eight cases, indicating the angiotropic feature of HAS. Etoh et al. (19) reported one HAS case without hepatic metastasis but with PVTT. In their study, five out of seven cases with venous tumor thrombus had hepatic metastasis, and two had no hepatic metastasis (**Figure 4**), indicating that tumor thrombus could be developed in a primary gastric tumor, invade peripheral veins, and further develop into PVTT. When elevated AFP is seen and mass is found in the liver on CT images, we need to be cautious in differentiating HAS from primary liver cancer. Liver biopsy and the gastroscope at this point would be crucial for diagnosis confirmation, clinical treatment, and the evaluation of prognosis.

The first-line treatment for HAS is radical surgery. Systemic chemotherapy is usually the choice for advanced-stage patients ineligible for surgery, and those with hepatic metastasis could be treated with transhepatic arterial chemotherapy and embolization. Monitoring serum AFP levels enables a timely detection of response or progression in gastric patients, and treatment plans could be made accordingly (20).

### Limitation of this study

Because of the small sample size, this study could not comprehensively reflect the imaging features of HAS, especially of various subtypes. The imaging findings of gastric adenocarcinoma are diverse. In this study, we did not set a control group with the CT findings of adenocarcinoma. Moreover, this study only covered CT images and no comparison with MR and other imaging techniques was carried out.

This study indicated the following characteristics of HAS: (1) HAS is often manifested as infiltrating gastric ulcers. On enhancement CT, it shows continuous progressive enhancement, prone to perigastric structures and LN metastasis. (2) More than 50% of HAS has an evident hepatotropic feature, prone to hepatic metastasis or tumor thrombus in the portal vein system. We should pay attention to its differentiation from primary liver cancer. (3) When serum AFP is elevated and intrahepatic/gastric lesions are found, we should differentiate hepatocellular carcinoma from HAS.

## Data availability statement

The raw data supporting the conclusions of this article will be made available by the authors, without undue reservation.

## Ethics statement

Written informed consent was obtained from the individual(s) for the publication of any potentially identifiable images or data included in this article.

## Author contributions

Conceptualization: YL, QY, and SZ. Data curation: YL, QY, and SZ. Formal analysis: QY, and SZ. Resources: QY and SZ. Writing—original draft: YL, QY and SZ. Writing—review and editing: YL. All authors contributed to the article and approved the submitted version.
